# Combined Use of Frameless Neuronavigation and In Situ Optical Guidance in Brain Tumor Needle Biopsies

**DOI:** 10.3390/brainsci13050809

**Published:** 2023-05-16

**Authors:** Elisabeth Klint, Johan Richter, Karin Wårdell

**Affiliations:** 1 Department of Biomedical Engineering, Linköping University, 581 85 Linköping, Sweden; 2 Department of Neurosurgery, Linköping University Hospital, 581 85 Linköping, Sweden

**Keywords:** five-aminolevulinic acid (5-ALA), image-guidance, laser Doppler flowmetry (LDF), microcirculation, MRI, neurosurgery

## Abstract

Brain tumor needle biopsies are performed to retrieve tissue samples for neuropathological analysis. Although preoperative images guide the procedure, there are risks of hemorrhage and sampling of non-tumor tissue. This study aimed to develop and evaluate a method for frameless one-insertion needle biopsies with in situ optical guidance and present a processing pipeline for combined postoperative analysis of optical, MRI, and neuropathological data. An optical system for quantified feedback on tissue microcirculation, gray–whiteness, and the presence of a tumor (protoporphyrin IX (PpIX) accumulation) with a one-insertion optical probe was integrated into a needle biopsy kit that was used for frameless neuronavigation. In Python, a pipeline for signal processing, image registration, and coordinate transformation was set up. The Euclidian distances between the pre- and postoperative coordinates were calculated. The proposed workflow was evaluated on static references, a phantom, and three patients with suspected high-grade gliomas. In total, six biopsy samples that overlapped with the region of the highest PpIX peak without increased microcirculation were taken. The samples were confirmed as being tumorous and postoperative imaging was used to define the biopsy locations. A 2.5 ± 1.2 mm difference between the pre- and postoperative coordinates was found. Optical guidance in frameless brain tumor biopsies could offer benefits such as quantified in situ indication of high-grade tumor tissue and indications of increased blood flow along the needle trajectory before the tissue is removed. Additionally, postoperative visualization enables the combined analysis of MRI, optical, and neuropathological data.

## 1. Introduction

Brain tumor needle biopsies are performed to retrieve tissue samples for neuropathological analysis to diagnose and tailor therapy. The general needle biopsy procedure is image-guided and utilizes preoperative planning, complication prediction, and intraoperative spatial feedback [1,2]. However, factors such as tumor growth, geometrical distortions, patient movement, and brain shifts decrease the pertinence of the preoperative images during surgery. Furthermore, a misalignment can occur between the preoperative images and the physical brain during registration for frameless procedures [3]. Consequently, there are risks of brain lesioning, hemorrhage [4,5], and sampling of non-tumor tissue. The latter yields inconclusive results [6,7], necessitating repeated needle insertions and, thus, increasing risks and extending the operation time.

Various optical techniques have been suggested for intraoperative feedback [8,9,10]. In needle biopsies, fluorescence induced by 5-aminolevulinic acid (5-ALA) [11,12] and, recently, sodium fluorescein (NaF) [13,14] has been shown to have clinical benefits by examining tissue samples under the microscope. However, as feedback is given after tissue sampling, non-diagnostic or even healthy tissue could be removed. Additionally, the interpretation of the fluorescence signal is not measured but depends solely on the individual surgeon’s subjective grading [11,12].

To quantify fluorescence, our group previously developed a spectroscopic probe system for detecting 5-ALA-induced fluorescence [15]. Furthermore, the system monitors changes in microcirculation and tissue gray–whiteness by using laser Doppler flowmetry (LDF) [16,17]. This setup was optimized for frame-based biopsy with the Leksell Stereotactic System (LSS) in a 20-patient cohort [18] and further integrated with an LSS-compatible biopsy needle [19].

The traditional biopsy procedure is based on a frame fixed to the skull and an attached device for the guidance of a biopsy needle through a burr hole trephination with mechanically set ‘stereotactic’ coordinates, which are calculated from preoperative images. Frameless methods relate to the skull by optical means but also rely solely on preoperative images. While studies have reported undistinguishable safety and efficacy for frame-based and frameless biopsy methods [20,21,22], some studies have also presented a reduction in the overall surgery time [20,23,24] and increased ease of use [25,26] for frameless biopsy methods. Implementing optical guidance in frameless neuronavigation biopsies could offer similar clinical benefits to those for frame-based methods, i.e., quantified in situ indication of high-grade tumor tissue and increased blood flow along the needle trajectory before the tissue is removed. Before these benefits can be utilized in a clinical setting, several additional steps need to be addressed. These steps include redesigning the probe system for integration with the frameless procedure to minimize the number of insertions and combining the optical data with the image information by using transformations into the same space.

The aim of this study was to develop and evaluate a method for MRI-based frameless brain tumor biopsies with in situ optical guidance and to present a data processing pipeline for combined postoperative analysis. The proposed method was implemented in a workflow that was experimentally evaluated on a phantom and in three patients undergoing needle biopsies.

## 2. Materials and Methods

**Figure 1:**
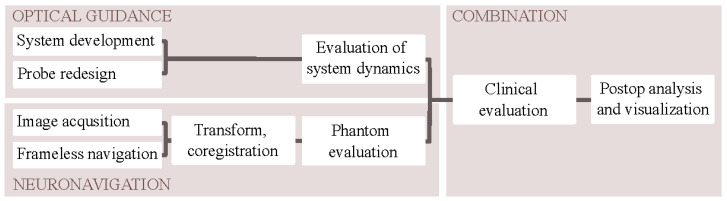
Block diagram of the methodology. Optical guidance and neuronavigation were evaluated separately before the combined clinical evaluation and postoperative visualization.

An overview of the different methodological components, optical guidance, neuronavigation, and their combination is presented in Figure 1.

### 2.1. Optical Guidance

#### 2.1.1. System Development

An in-house optical system that was previously described in [27] and that combined LDF (PF 5010, Perimed AB, Stockholm, Sweden) and 5-ALA-induced fluorescence was updated. Briefly, tissue *Perfusion* (i.e., microvascular blood flow) and total light intensity (*TLI*, corresponding to gray–white matter changes) were continuously and simultaneously recorded with point-wise tissue fluorescence spectra. The in-house-developed LabView software (2019, National Instruments, Austin, TX, USA) controlled the fluorescence acquisition settings and presented signals in real time. The ratio, *R*, between tissue autofluorescence (max intensity between wavelengths: (λ) 470–550 nm) and the intensity at 635 nm was calculated [28]. A peak at 635 nm represented protoporphyrin IX (PpIX) accumulation, indicating high-grade tumor tissue. For further details of the system, see Appendix A.

#### 2.1.2. Probe Redesign

A fiber optical probe (∅ 1.7 mm, length: 270 mm, optical fibers: two ∅ 200 μm, numerical aperture (NA): 0.22 and four ∅ 125 μm, NA: 0.37) was designed to fit into the outer cannula of the frameless navigation biopsy needle (∅ 2.2 mm, length: 270 mm, Passive Biopsy Needle Kit, Medtronic Inc., Minneapolis, MN, USA). For each disposable biopsy kit, an aperture was made at the tip of the outer cannula to allow forward-looking tissue measurements. The tips of both the outer cannula and probe were slightly rounded and polished until smooth. It was ascertained that the functional length of the biopsy needle was kept, and the ‘probe-needle kit’ was tested together to ensure that their respective functionalities were intact.

#### 2.1.3. Evaluation of System Dynamics

The fluorescence module was tested for laser powers, *P*, between 5 and 20 mW, and pulse lengths, *PL*, of 200, 400, 600, and 800 ms were used to determine the optimal default settings. The evaluation was performed both on a static reference and, as presented in [27], the ventral forearm skin of a healthy volunteer treated with a fluorescent drug (METVIX^®^ 160 mg/g, PhotoCure ASA, Oslo, Norway, Ethics no. M139-07/T83-09) [29]. The LDF signals were tested against a standard microsphere solution (PF1001 Motility, Perimed AB) and on the skin. The simultaneous acquisition was evaluated to investigate the interference between the fluorescence and LDF signals.

Before each use, measurements on the static reference and in the Motility were acquired and checked to determine if they were within acceptable ranges. The probe and the static reference were sterilized according to the Sterrad^®^ protocol.

### 2.2. Neuronavigation

#### 2.2.1. Image Acquisition

Preoperative magnetic resonance (MR) images were acquired on Siemens 3T scanners (Prisma or Skyra, Siemens Healthineers, Erlangen, Germany). T_1_-weighted (T_1_w) images with and without gadolinium (Gd) contrast enhancement, as well as T_2_w and T_2_w-FLAIR images, were acquired according to the clinical navigation protocol. The acquisition details of each MR sequence were as follows:

T_1_w 3D gradient echo before and after Gd (T_1_wGd) administration: voxel size: 1.0 × 1.0 × 1.0 mm^3^, FOV: 256 × 176 mm^2^, t_TE_ = 2.26 ms, t_TR_ = 2300 ms, t_TI_ = 900 ms, GRAPPA 2 (ref lines 24), Gd dose: 16–17 mL (Dotarem, 279 mg/mL);

T_2_w 3D spin echo: voxel size: 0.9 × 0.9 × 1.0 mm^3^, FOV: 240 × 176 mm^2^, t_TE_ = 407 ms, t_TR_ = 3200 ms, GRAPPA 2 (ref lines 24);

T_2_w-FLAIR 3D spin echo: voxel size: 1.0 × 1.0 × 1.0 mm^3^, FOV: 256 × 176 mm^2^, t_TE_ = 388 ms, t_TR_ = 5000 ms, t_TI_ = 1600 ms, GRAPPA 3 (ref lines 24).

Postoperatively, either computed tomography (CT, SOMATOM Definition Edge, Siemens Healthcare, Germany) or T_1_w 3D MR images (Prisma or Skyra) were acquired to detect potential hemorrhages and confirm the biopsy location. The final biopsy position was defined as the center of the observed cavity.

#### 2.2.2. Frameless Navigation

The preoperative images were downloaded and coregistered on a frameless neuronavigation system (StealthStation S8, Medtronic Inc., USA). The navigation system was also used for trajectory and target planning. Registration between the images and the patient’s physical anatomy was performed by tracing the scalp; then, the trajectory was locked. Errors between the trace and the preoperative images (registration error) and between the locked and planned trajectories (targeting error) were noted. The instruments were held in place by an AutoGuide^®^ (Medtronic Inc., USA). The coordinates of the instruments in the navigation space were logged through the developer mode in the navigation system. Images, metadata, and coordinate points were exported from the navigation system for further analysis in Python.

#### 2.2.3. Coordinate Transform and Image Coregistration

**Figure 2:**
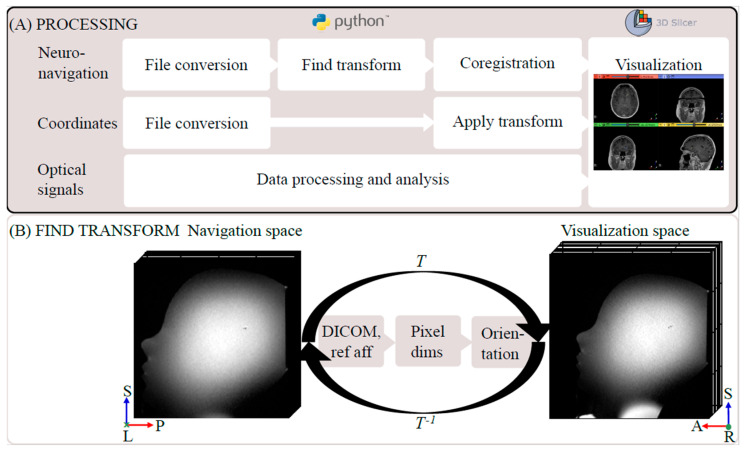
Processing pipeline including the coordinate transform. (**A**) Processing pipeline from export to visualization of the images used for neuronavigation, the intraoperative MR coordinates, and the optical signals. (**B**) Phantom example of the ‘Find transform’ step between the navigation and visualization space, which utilized the DICOM information and took other reference volumes into account. Image orientation R: right, L: left, A: anterior, P: posterior, S: superior. Ref: reference.

The pipeline from the acquired DICOM images to the combined visualization is presented in Figure 2A. In Python (v.3.8), the images were converted to the NIfTI-format by using a wrapper function for dcm2niix [30]. Then, the affine transform, *T*, was calculated from the navigation—via DICOM—to the visualization space by utilizing the q- and s-form matrices from the DICOM tags. Additionally, other reference spaces in the navigation system were taken into account (e.g., preoperative CT images), and *T* was corrected for variations in pixel size and coordinate system orientation. The resulting matrix had twelve degrees of freedom of the form

(1) $$T= [\begin{array}{cc} \begin{array}{cc} r_{11} & r_{12}\\ r_{21} & r_{22} \end{array} & \begin{array}{cc} r_{13} & t_{x}\\ r_{23} & t_{y} \end{array}\\ \begin{array}{cc} r_{31} & r_{32}\\ 0 & 0 \end{array} & \begin{array}{cc} r_{33} & t_{z}\\ 0 & 1 \end{array} \end{array}],$$

where r*_ij_* (*i, j* = {1..3}) constitute the linear map (rotation, scaling, and shearing in three spatial dimensions), and t*_k_* (*k* = {*x*, *y*, *z*}) are the translational components (translation in three spatial dimensions). For a schematic illustration of the resulting transform, see Figure 2B.

The pre- and postoperative images were coregistered in Python by using affine linear registration in Advanced Normalization Tools (ANTs) [31] by utilizing the Nipype framework [32]. In the postoperative images, the final entry and biopsy locations were identified by the operating neurosurgeon (J.R.) and transformed into the patient-specific visualization space. Based on these final locations, the preoperative image coordinates of each optical measurement were projected onto the final trajectory by using the Euclidian distance. The final coordinates were superimposed on the preoperative images in the visualization space (3D Slicer, v5.2.1 [33]).

#### 2.2.4. Phantom Evaluation

A low-cost MRI-compatible phantom was constructed by anchoring a ball (∅ 3 mm, mimicking a tumor) in the central region of a plastic head mold. After silicone sealing, the phantom was filled with saline solution, and T_1_w and T_2_w 3D MR images were acquired (see the example in Figure 2B). In these images, a trajectory was planned with the ball as the intended target. Registration was performed, the trajectory was locked, and errors were noted. A biopsy needle kit was secured and inserted into the target. Coordinates for points along the trajectory were logged. The procedure—from registration to reaching the target—was repeated three times.

The transformation matrix *T* was calculated and verified by applying *T* to both random and unit vector points in the navigation space and comparing the resulting coordinates in the visualization space. The matrix inverse (*T^−^*^1^) was used to address the inverse problem.

### 2.3. Combined Evaluation

**Figure 3:**
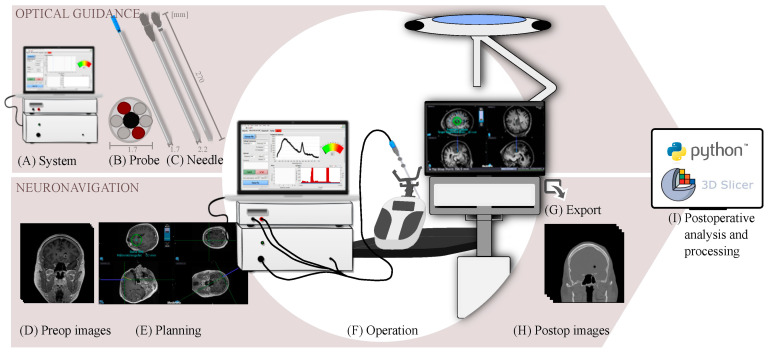
Clinical workflow for preparation, data acquisition, and export in a clinical setting for frameless MRI-based neuronavigation with in situ optical guidance. (**A**) An optical measurement system and (**B**) probe integrated into the (**C**) modifised biopsy needle were tested and prepared for surgery. (**D**) Preoperative images were acquired, and (**E**) trajectory planning was performed before surgery. (**F**) During operation, optical signals were displayed in real time and navigation coordinates were logged. (**G**) After the procedure, the images, metadata, and optical signals were exported. (**H**) Postoperative images were acquired, and finally, (**I**) analysis was performed for multiparametric visualization.

The method of frameless MRI-based navigation with optical guidance was implemented in three patients undergoing brain tumor needle biopsies; see the schematic illustration of the clinical workflow in Figure 3.

#### 2.3.1. Patients

Three patients (mean age: 66 years, range: 53–78, one female) planned for frameless needle biopsy at Linköping University Hospital within one month in 2022 were included in the study. The patients had suspected high-grade tumors, as indicated by the contrast enhancement in MRI. This study was carried out in accordance with the Declaration of Helsinki. Study approval was granted by the Swedish Ethical Review Authority (EPM 2020-01404), and written informed consent was obtained from all patients.

#### 2.3.2. Frameless Neuronavigation with Optical Measurements

An oral dose of Gliolan^®^ (20 mg/kg, Medac GmbH, Wedel, Germany, 5-ALA) was given to the patient two to three hours before general anesthesia according to the clinical routine for fluorescence-guided resection [9].

After trajectory planning and registration, a 3.2 mm burr hole was made in the skull, and the dura was opened. Before the investigational optical probe was secured inside the outer cannula of the biopsy needle kit, a functional test on the sterilized static reference was made. The probe–needle kit was fastened to the AutoGuide^®^, the trajectory was locked, and errors were noted. In situ measurements of brain tissue *Perfusion*, *TLI*, and fluorescence were taken in steps of a few millimeters, as indicated in the navigation software, and shown to the surgeon in real time. The probe was held still for 5–15 s at each point to ensure that the LDF measurements were without movement artifacts. If increased *Perfusion* values were detected, the question of whether to continue or choose another trajectory was posed. The navigation coordinates of each measurement point were logged.

The site with the highest 5-ALA-induced fluorescence peak was identified. If no contraindications were found, the probe–needle kit was adjusted so that the biopsy sampling window coincided with the identified PpIX peak site. Then, the probe was replaced by the inner cannula of the biopsy needle, and the tissue was sampled and sent for a routine intraoperative smear. The patient was prepared for the end of surgery, and the waiting time from tissue sampling to a response from the clinical pathologist was noted.

Within two weeks of surgery, a final diagnosis was given according to the CNS WHO 2021 classification [34].

#### 2.3.3. Postoperative Analysis and Visualization

The mean and standard deviation of the *Perfusion* and *TLI* signals for each measurement position were calculated over 5–10 s intervals. Additionally, the fluorescence spectra and *R* were plotted along the trajectory.

The transform *T* was calculated and applied to the navigation coordinates. The coregistered images, analyzed optical signals, pre- and postoperative needle biopsy trajectories, and transformed coordinates were imported into 3D Slicer (Figure 2A). The results of the coregistration were visually assessed by the operating neurosurgeon. The Euclidian distance between the pre- and postoperative coordinate points in the coregistered images was calculated.

## 3. Results

The optical and navigation subsystems were evaluated separately on the references, skin, and a phantom. Then, the combined use of frameless MRI-based neuronavigation and in situ optical feedback was successfully evaluated in the three patient cases. An overview of the proposed clinical workflow can be seen in Figure 3. The postprocessing pipeline (Figure 2A) from DICOM images to combined visualization took approximately 30 min and was automated.

### 3.1. Optical System Dynamics

**Figure A1:**
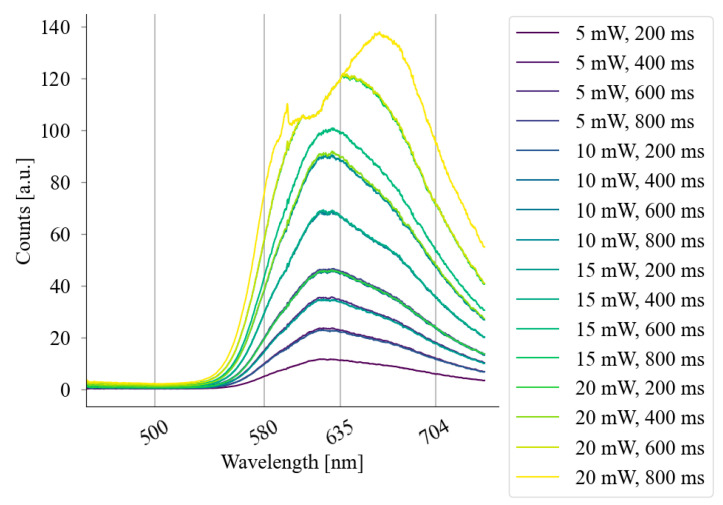
Fluorescence counts for various laser powers (5–20 mW) and pulse lengths (200–800 ms) against a static reference.

**Figure A2:**
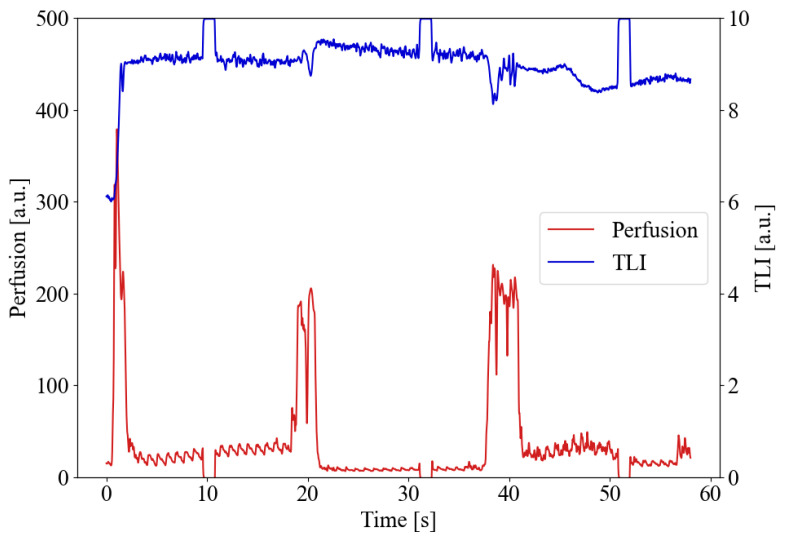
*Perfusion* and *TLI* signals from three measurement points recorded on brain tissue. The large (200–400 counts) irregular spikes are artifacts caused when moving the probe from one position to the next. The square features (occurring around 10, 32, and 52 s) were caused by the fluorescence acquisition, namely, when the near-UV laser was on.

The fluorescence and LDF signals on the static reference, skin, and Motility were found to be within acceptable bounds. For the fluorescence, the counts varied from none to a saturated detector for *P* = 5–20 mW and *PL* = 200–800 ms. To minimize photobleaching while maintaining a suitable excitation energy and maximizing the use of system dynamics, the default settings were 10 mW and 400 ms (Appendix A, Figure A1). The fluorescence signal was unaffected by the LDF laser due to a cut-off filter. However, transient pulses (3 × *PL*, default: 1.2 s) were seen in the LDF signal when the fluorescence laser was on (Appendix A, Figure A2).

### 3.2. Phantom Neuronavigation and Transform

**Figure 4:**
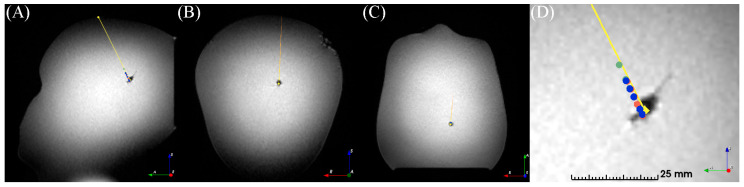
Registration and transformation of three consecutive frameless navigation procedures on a low-cost MRI head phantom. (**A**) Oblique sagittal, (**B**) coronal, and (**C**) axial slices of the T_1_w image volume. (**D**) Zoom of the target area in the sagittal view. (**A**–**D**) Diamonds and lines indicate thepreoperatively planned trajectory, circles indicate the intraoperative measurement points. Image orientation R: right, A: anterior, S: superior.

For the three registrations of the in-house phantom, the coordinate points varied by 1.1 ± 0.3 mm between the planned and logged coordinates. The observed errors were 1.4–1.5 mm for registration and 1.1–1.6 mm for targeting. The coordinates are presented in the visualization space in Figure 4.

The predefined points in the navigation and visualization spaces for the phantom showed negligible errors when using the automated transform.

### 3.3. Patient Cases

**Figure 5:**
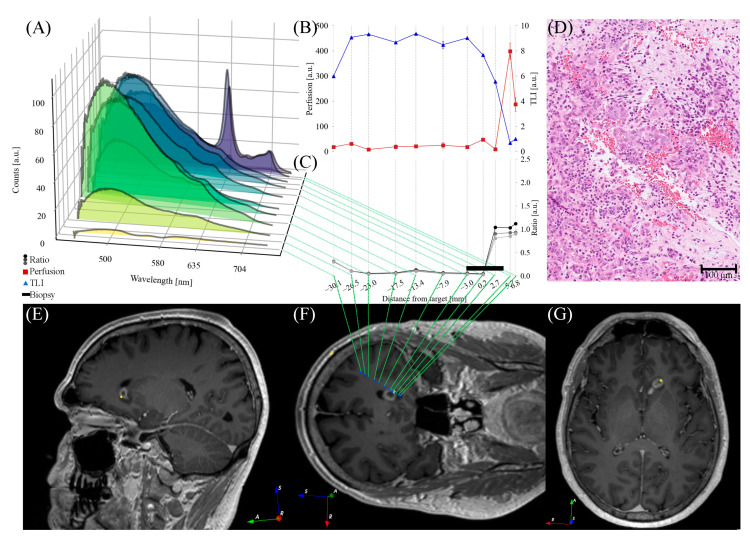
Optical signals ((**A**) fluorescence spectra, (**B**) *Perfusion* and *TLI* signals, and (**C**) fluorescence ratio *R*) for each measurement position along the trajectory for patient 1. (**D**) Example of an H&E-stained section from the neuropathological analysis. (**E**) Oblique sagittal, (**F**) coronal, and (**G**) axial T_1_wGd image that is tilted to capture the postoperative trajectory. (**E**–**G**) Blue circles indicate measurement positions, yellow diamonds indicate final entry and biopsy positions. Image orientation R: right, A: anterior, S: superior.

**Figure 6:**
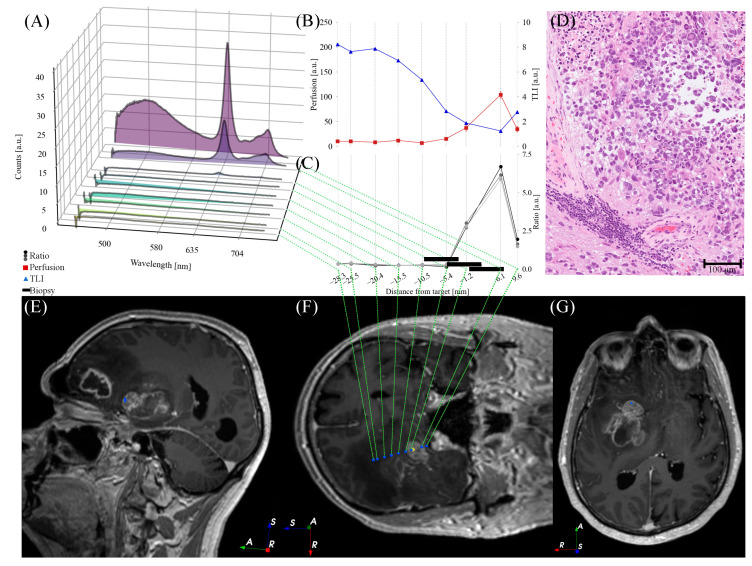
Optical signals ((**A**) fluorescence spectra, (**B**) *Perfusion* and *TLI* signals, and (**C**) fluorescence ratio *R*) for each measurement position along the trajectory for patient 2. (**D**) Example of an H&E-stained section from the neuropathological analysis. (**E**) Oblique sagittal, (**F**) coronal, and (**G**) axial T_1_wGd image that is tilted to capture the postoperative trajectory. (**E**–**G**) Blue circles indicate measurement positions, yellow diamonds indicate final entry and biopsy positions. Image orientation R: right, A: anterior, S: superior.

**Figure 7:**
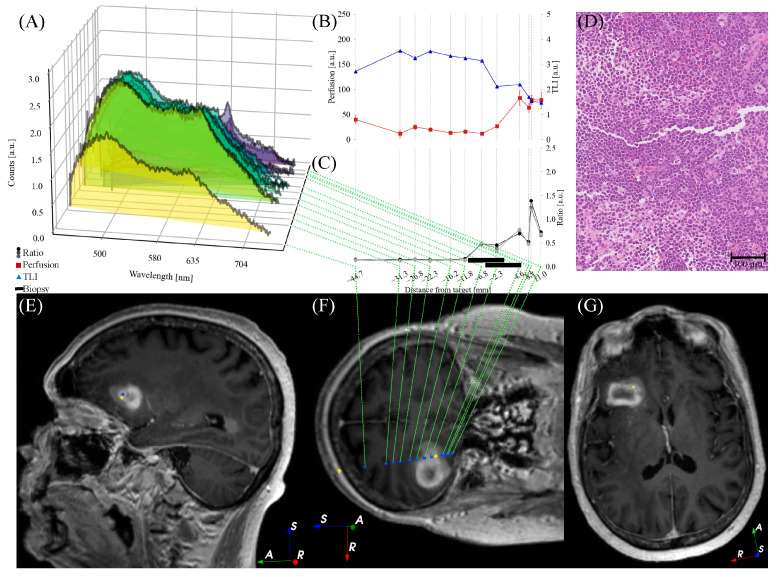
Optical signals ((**A**) fluorescence spectra, (**B**) *Perfusion* and *TLI* signals, and (**C**) fluorescence ratio *R*) for each measurement position along the trajectory for patient 3. (**D**) Example of an H&E-stained section from the neuropathological analysis. (**E**) Oblique sagittal, (**F**) coronal, and (**G**) axial T_1_wGd image that is tilted to capture the postoperative trajectory. (**E**–**G**) Blue circles indicate measurement positions, yellow diamonds indicate final entry and biopsy positions. Image orientation R: right, A: anterior, S: superior.

*Perfusion*, *TLI*, and PpIX fluorescence were presented in situ in 1–14 mm steps along the trajectory, with shorter steps in the target region (Figure 5, Figure 6 and Figure 7). PpIX peaks were found between 1 mm ahead of (Figure 6) and 11 mm beyond (Figure 7) the predefined targets in the preoperative MRI. Biopsy samples were collected in the tissue volume corresponding to the largest peak (Figure 5, Figure 6 and Figure 7A) and ratio (Figure 5, Figure 6 and Figure 7C) without increased *Perfusion*. A neuropathologist confirmed the samples as tumor tissue within 50–60 min. Increased *Perfusion* levels (above 100 a.u.) were detected at three points (Figure 5B and Figure 6B), but neither was high enough to result in a trajectory change.

**Figure A3:**
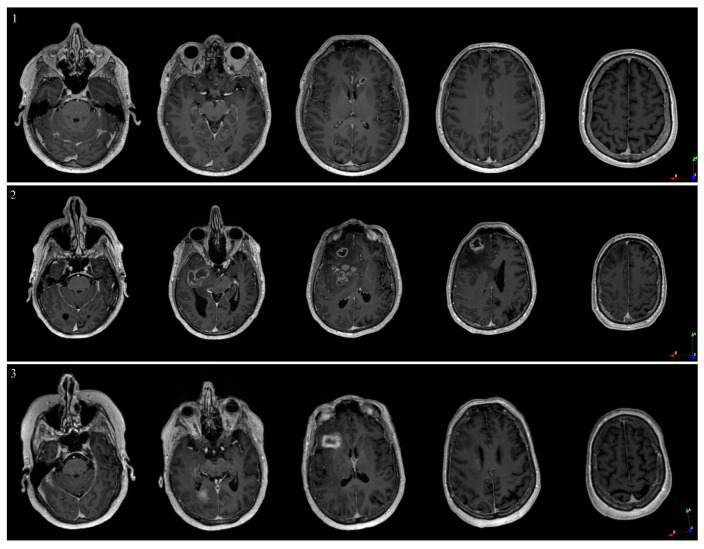
Sample image slices from the T_1_wGd image volume used for neuronavigation for patient 1 (top), 2 (middle), and 3 (bottom). Image orientation R: right, A: anterior, S: superior.

The registration errors were 1.5–1.9 mm and the trajectory targeting errors were 0.1–0.5 mm. For additional slice examples of the preoperative navigation images, see Figure A3 in Appendix B. The default settings for the optical system were used, except for one patient in whom *P* was increased to 15 mW due to the consistently low fluorescence signals (Figure 7A). The total duration of the functional tests, optical measurements, and annotations was 7–9 min.

Postoperative CT or MR images were acquired within 12 h of surgery. The images confirmed the biopsy sample locations and did not indicate any hemorrhages. The final CNS WHO 2021 diagnoses and grades can be found in Table 1, along with other patient-specific details. For examples of hematoxylin- and eosin-stained histological images, see Figure 5, Figure 6 and Figure 7D.

The combined visualization illustrated the final coordinate points and indicated a shift of 2.5 ± 1.2 mm between the pre- and postoperative locations.

## 4. Discussion

An investigational one-insertion optical probe system integrated into the frameless MRI-based neuronavigation brain tumor needle biopsy procedure was presented. This translation toward clinical use allows real-time optical feedback while minimizing the number of insertions. To increase the understanding and interconnection between the modalities, the coordinates of the optical measurements were collected and mapped to the corresponding points in the pre- and postoperative images. The automated pipeline for postoperative analysis allows combined visualization of multiparametric data. The methodology offers benefits such as in situ measurements of tissue characteristics before sampling and postoperative visualization for multiparametric analysis. To our knowledge, no other study has combined optical signals, intraoperative MR coordinates, postoperative imaging, and neuropathological results to achieve intraoperative real-time feedback and support postoperative evaluation and verification with the resolution presented herein.

Figure 5, Figure 6 and Figure 7 illustrate the heterogeneity in the task at hand. Variations are seen both within patients (e.g., autofluorescence intensity at different positions) and between patients (e.g., the overall shape of the measured fluorescence response, Figure 5, Figure 6 and Figure 7A). This is also seen in the varying fluorescence ratios (Figure 5, Figure 6 and Figure 7C) and the neuropathological images (Figure 5, Figure 6 and Figure 7D). As the tissue fluorescence and *Perfusion* values are presented in arbitrary units, further comparison between the patients is discouraged. The overall shapes of the *TLI* curve (Figure 5, Figure 6 and Figure 7B) are similar as the probe-needle kit goes through the cortex and white matter (slightly brighter, i.e., higher *TLI* values) and reaches tumor tissue (darker, i.e., lower *TLI* values).

The overall goal of the needle biopsy procedure is to pose a diagnosis for tailoring further treatment. Thus, in cases in which the neurosurgeon determined the tissue material in the first biopsy position to be insufficient in terms of volume, the needle was moved to a new position, and additional tissue material was sampled.

### 4.1. In Situ Optical Feedback on Tissue Characteristics

#### 4.1.1. Indication of Tumor Tissue

PpIX fluorescence shows selective accumulation in neoplastic cells [35] and is routinely used during resection for the identification of high-grade tumor tissue [9,10,36] and lymphomas [37,38]. Accumulation has also been indicated for meningiomas [39,40,41] and metastases [36,42,43]. If no PpIX fluorescence peak is registered, tissue collection falls back to the standard method that relies on preoperative images, and increased *Perfusion* and *TLI* are still reported.

Other groups have also suggested methods for intraoperative feedback of tissue [11,12,13,14,44,45]. These techniques aim to replace intraoperative pathology through optical feedback, but as the tissue is placed under the microscope after it has been sampled, further benefits are to be gained. A probe system, such as the one presented here, allows for in situ feedback in millimeter steps before the tissue is sampled, both in the sampling position and along the needle trajectory. Other optical markers have been explored. For example, Desroches et al. introduced a Raman probe for measurements in vivo [46]. Raman spectroscopy is a label-free technique based on the molecular vibrations of the tissue with the potential for differentiating tumor and non-tumor tissue [47,48,49]. However, the signals require extensive processing and interpretation, thus currently limiting their intraoperative clinical pertinence. In addition, NaF has been suggested for visualizing tumor tissue [8,14]. The cost of NaF is often mentioned as the main advantage of NaF compared to 5-ALA; however, as Millesi et al. stated, in-house pharmaceutical production of 5-ALA markedly decreases these costs [12].

So far, the probe system introduced in this study has been adapted for the StealthStation and 5-ALA, but it can be modified to fit other setups. Modifying the probe dimensions allows for integration with the LSS frame [19] and use during tumor resection [50].

#### 4.1.2. Addressing the Risk of Hemorrhage

During the biopsy procedure, hemorrhage can occur either when the biopsy needle kit is pushed along the trajectory or when the needle cuts the tissue during sampling. Even though the overall risk of hemorrhage is difficult to eliminate, increased *Perfusion* can indicate areas in which to take extra care and those that should be avoided altogether. In one patient, increased *Perfusion* coincided with a clear PpIX peak, indicating that this point (~6 mm beyond the preoperative target, Figure 5B) was likely tumor tissue, but should still be avoided to decrease the risk of hemorrhage.

Both Wilson et al. [51] and Ramakonar et al. [52] illustrated the potential to detect blood vessels through the side-cutting window of a biopsy needle by using optical spectroscopy and optical coherence tomography probes, respectively. These probes do not, however, address the microcirculation during insertion.

### 4.2. Navigation in Neurosurgery

MRI-based navigation enables planning and feedback, which are crucial to the procedure. However, one could argue that the images are obsolete at the moment in which the patient is moved from the MR scanner due to, e.g., changed head position, intubation, and fluid regulation. Even in the phantom simulation, though movement during image acquisition, brain shift, and tumor growth were eradicated, a discrepancy was observed in the resulting coordinate points for the three registrations. This discrepancy was most likely due to errors in registration and targeting, which was supported by their order of magnitude. Other studies reported errors in the range of 0.1–3.3 mm [53,54], which corroborated our findings for both the phantom and patient cases. In the patient cases, the discrepancy between the pre- and postoperative positions was most likely a combination of registration and targeting errors, condition changes, including brain shifts and fluid leakage between imaging and operation, and interpolations made during image registration and transform calculation. Therefore, we stress the importance of using postoperative images to verify the actual trajectory used during the procedure, especially in the interest of the comparison of modalities. Further, as additional MRI sequences, such as quantitative MRI, are developed to aid in planning, it is important to keep this misalignment in mind.

Other groups have illuminated the benefit of combining 5-ALA-induced fluorescence and MRI. For example, Giordano et al. [44] and Roberts et al. [55] compared contrast enhancement in intraoperative and preoperative MRI, respectively, with fluorescence under a blue light microscope during resection. Both studies found a strong relationship between fluorescence and contrast enhancement.

### 4.3. Limitations and Outlook

The different modalities used herein present data with different resolutions. For example, the MR protocol specifies isotropic 1 mm^3^ voxels, a navigation system with submillimeter precision, optical measurements with an approximate 1 mm look-ahead distance [56,57], and a biopsy needle window of 8 mm in length; neuropathology is performed on submillimeter tissue sections. With changing conditions and varying registration errors reported in the literature, the final resolution should be stated with caution. 

To evaluate the methodology further, a larger patient cohort is needed. Hence, no conclusions were drawn regarding the mean registration error, size of the brain shift, shortened procedure time, or reduction in the associated risks. Therefore, currently, more data are being collected to analyze these parameters in a follow-up study. Present investigations also include the potential of other MRI sequences for giving feedback on the tissue microstructure.

## 5. Conclusions

In conclusion, the presented in situ optical guidance method gives feedback on the tissue along the biopsy’s trajectory before the tissue is removed. The quantified optical signals represent the tissue microcirculation, gray–whiteness, and PpIX accumulation. Integration into the frameless MRI-based needle biopsy procedure allows further postoperative analysis by relating the signals to coordinates in the pre- and postoperative images.

## Figures and Tables

**Table 1:** Image acquisition parameters, neuronavigation errors, timeframes, final neuropathological diagnoses, and Euclidian distance for the three patients. CNS: central nervous system, IDH: isocitrate dehydrogenase, intraop: intraoperative, WHO: World Health Organization.

	Patient		
Parameter	1	2	3
*Image Acquisition*			
Reference space	MR DICOM	CT	MR DICOM
Pixel spacing [mm]	1 × 1 × 1	1 × 0.977 × 0.977	1 × 1 × 1
Field of view [mm]	192 × 256 × 256	176 × 256 × 256	176 × 256 × 256
Acquisition time [min]	21	21	21
Postoperative image type	CT	CT	MR
*Neuronavigation Errors*			
Registration [mm]	1.9	1.5	1.6
Targeting [mm]	0.1	0.2	0.5
*Time*			
Optical measurement [min]	7	7	9
Intraop diagnosis [min]	50	60	60
*Final Diagnosis*			
CNS WHO 2021	High-grade astrocytoma, IDH-wildtype	Glioblastoma, IDH-wildtype, grade 4	Primary diffuse large B-cell lymphoma of the CNS
*Analysis (pre- vs. postop)*			
Euclidian distance [mm]	3.1 ± 0.48	3.3 ± 1.2	1.3 ± 0.25

## Data Availability

The data are not publicly available due to privacy regulations.

## Appendix A

*Optical Measurement System*

The dual-laser optics system presented in this study consisted of a commercial LDF unit (λ = 780 nm, *P* = 2 mW, PF 5010, Perimed AB, Sweden) and a fluorescence module that was constructed in-house. The fluorescence module consisted of a near-UV laser (λ = 405 nm, ≤40 mW, Z-laser GmbH, Germany) and a CCD spectrometer (AVASpect-ULS2048L-EVO, Avantes, BV, The Netherlands). Wavelength calibration was carried out for the spectrometer. The two modules were linked through a data acquisition (DAQ) card (NI USB-6211, Nation Instruments, Austin, TX, USA).

A bandpass filter (long-pass, cut-on: λ = 455 nm, Newport, Newport Corporation, USA, and short-pass, cut-off: λ = 750 nm, Edmund Optics, Edmund Optics Inc., Barrington, NJ, USA) blocked the Rayleigh scattered light from the incident laser beams from reaching the spectrometer.

*Signal Acquisition*

Each fluorescence measurement consisted of one dark spectrum (laser off) followed by three light spectra (laser on). The dark spectrum was subtracted from each light spectrum to remove the influence from the surrounding light.

The fluorescence acquisition settings could be set to laser powers between *P* = 5 and 20 mW; the pulse lengths were *PL* = 50–1000 ms, and there were 1–1000 spectra within the wavelength interval λ of 450–750 nm. The default settings were 10 mW and 400 ms (Figure A1), resulting in an excitation energy of 4 mJ. The optional settings included auditory feedback as the ratio *R* exceeded a user-specified threshold. The heart rate could be calculated from the small pulses in the *Perfusion* signal (Figure A2).

## Appendix B

*Imaging in Neurosurgery*
